# Sleeve Gastrectomy and Cholecystectomy are Safe in Obese Patients with Asymptomatic Cholelithiasis. A Multicenter Randomized Trial

**DOI:** 10.1007/s00268-022-06557-2

**Published:** 2022-04-10

**Authors:** Tamer A. A. M. Habeeb, Mohammad Kermansaravi, Mariano Eduardo Giménez, Mallikarjuna N. Manangi, Hosam Elghadban, Samar A. Abdelsalam, Abd-Elrahman M. Metwalli, Muhammad Ali Baghdadi, Abdelrahman A. Sarhan, Adel Mahmoud Moursi, Ahmed K. El-Taher

**Affiliations:** 1 Department of General Surgery, Faculty of Medicine, Zagazig University, Zagazig, Egypt; 2 Department of Surgery, Minimally Invasive Surgery Research Center, Division of Minimally Invasive and Bariatric Surgery, Rasool-E Akram Hospital, Iran University of Medical Sciences, Tehran, Iran; 3 Department of General and Minimally Invasive Surgery, University of Buenos Aires, Buenos Aires, Argentina; 4 Department of General Surgery, Bangalore Medical College and Research Institute, Bengaluru, India; 5 Department of General Surgery, Mansoura University, Mansoura, Egypt; 6 Family Medicine Department-Faculty of Medicine, Zagazig University, Zagazig, Egypt

## Abstract

**Background:**

Obesity is a severe health problem. Gallstones may symptomatize after sleeve gastrectomy (SG). Concomitant laparoscopic cholecystectomy (LC) with SG is controversial. The effects of SG and LC versus delayed LC following SG in obese patients with asymptomatic gallbladder stones were evaluated.

**Methods:**

A randomized trial of 222 morbidly obese patients with gallbladder stones divided them into two equal groups: SG + LC and SG-only. This multicenter study conducted from January 2016 to January 2019.

**Results:**

Except for operative time and postoperative hospital stay, there was no statistically significant difference between LSG + LC group and SG group (*P* < 0.001). In SG + LC group, LC added 40.7 min to SG, three patients (3%) required conversion, early postoperative complications occurred in 9 cases (9/111, 9%), three cases required re-intervention (3%). In SG group, the complicated cases required LC were 61 cases (61/111, 55%). Acute cholecystitis (26/61, 42.7%) was the most common gallstone symptoms. Most complicated cases occurred in the first-year follow-up (52/61, 85%). In the delayed LC group (61 patients), operative time was 50.13 ± 1.99 min, open conversion occurred in 2 cases (2/61, 3.2%), early postoperative complications occurred in four patients (4/61, 6.4%) and postoperative re-intervention were due to bile leaks and cystic artery bleeding (2/61, 3.2%).

**Conclusions:**

SG with LC prolongs the operative time and hospital stay, but the perioperative complications are the same as delayed LC; LC with SG minimizes the need for a second surgery. Concomitant LC with SG is safe.

## Introduction

Obesity is a global problem, according to the World Health Organization (WHO) [1]. Thousands of bariatric surgeries (BS) are performed yearly [2]. BS helps patients lose weight, corrects obesity-related issues and enhances life quality [3]. Sleeve gastrectomy (SG) evolved from a stepwise duodenal switch step to a major restrictive bariatric operation [4]. Gallbladder stones occur in up to 20% of the general population. Gallbladder stones are up to five times more common in obese people than in healthy people [5–7].

This study compares perioperative complications following SG + LC versus delayed LC after SG. Also, we aimed to detect the incidence and risk factors of symptomatic gall stones in SG cases with no concomitant LC.

## Material and methods

A randomized trial was planned in our university hospitals' bariatric surgical units from January 2016 to January 2019. Two hundred twenty-two obese patients with asymptomatic gallbladder stones were divided into two equal groups: SG with concomitant LC group and SG without concomitant LC group. The SG group without concomitant LC was followed up after surgery to assess the prevalence of symptomatic gallbladder stones requiring surgery and subsequently delayed LC complications. The sample size was computed as 222 (111 in each group) according to the previous literature [8]. Patients who chose odd numbers belonged to SG with concomitant LC group, while those who chose even numbers belonged to SG without concomitant LC group.

Candidates for bariatric surgery who met the National Institutes of Health (NIH) consensus criteria for the management of morbid obesity [9] (BMI 40 or 35 kg/m^2^ with comorbidities linked to obesity) and who had a preoperative ultrasound and completed the 2-year follow-up were included in the study. Exclusion criteria were ≤ 18 years, history of cholecystectomy or bariatric operation, a cognitive vulnerability, drug addicts, unsuitable for general anesthesia, patients with calcular obstructive jaundice, cases with problematic exposure of right upper quadrant or liver cirrhosis found at surgery and cases lost during follow-up.

Weight loss results are given as a % of excess weight loss (%EWL), with a BMI of 25 kg/m2 considered optimal [10]. Postoperative morbidity was evaluated by the Clavien and Dindo classification [11].

### Perioperative technique

In three years, five bariatric surgeons with a same technique completed all surgeries in our hospitals' multicenters, ensuring bariatric and biliary surgery basics. SG was done using conventional steps [12]. Following SG, the gallbladder was examined for cholecystectomy. We used bariatric incisions for LC. Patients consumed water the day after surgery. Protons pump inhibitor 40 mg once daily and Clexane 80 IU subcutaneously for thrombotic prophylaxis. After discharge, all patients were followed for 1, 3, 6, 12 and 24 months by telephone or in outpatient clinic.

### Statistical analysis

SPSS (version 20.0, Armonk, NY: IBM Corp) was used to analyze the collected data. Continuous variables have a mean and an SD (SD). Categorical variables were expressed as percentages. Statistics were evaluated using an independent *t*-test between groups and a repeated measure ANOVA with paired *t*-tests within each group, whereas qualitative data were evaluated using a Chi-square test (2). In this study, regression analysis was used to identify independent components. The symptom-free survival rate was calculated using Kaplan–Meier. *P* values ≤ 0.05* and ≤ 0.001** were considered statistically significant.

## Results

**Fig. 1:**
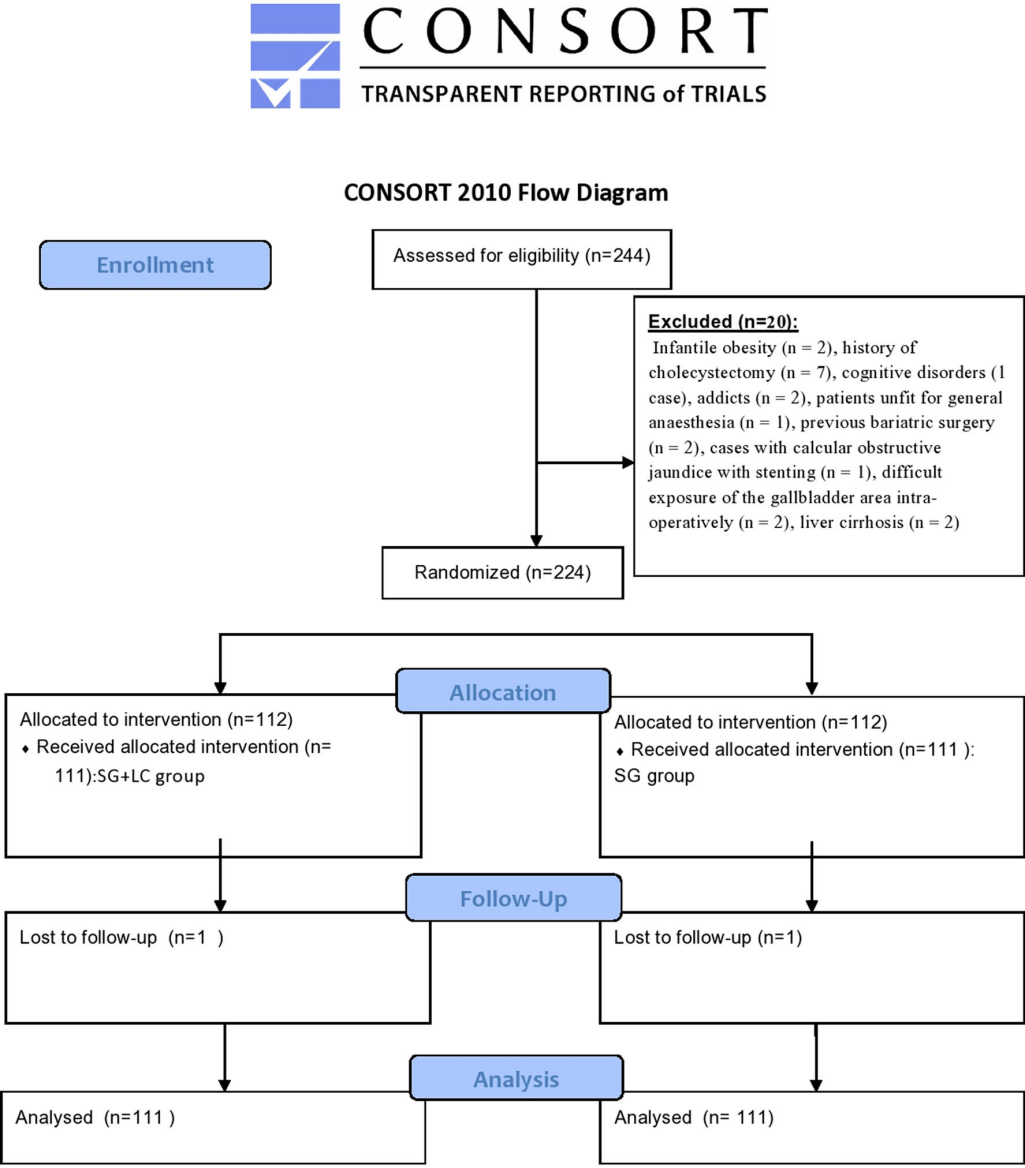
Flowchart

A flowchart evaluated inclusion and exclusion of patients (Fig. 1).

**Fig. 2:**
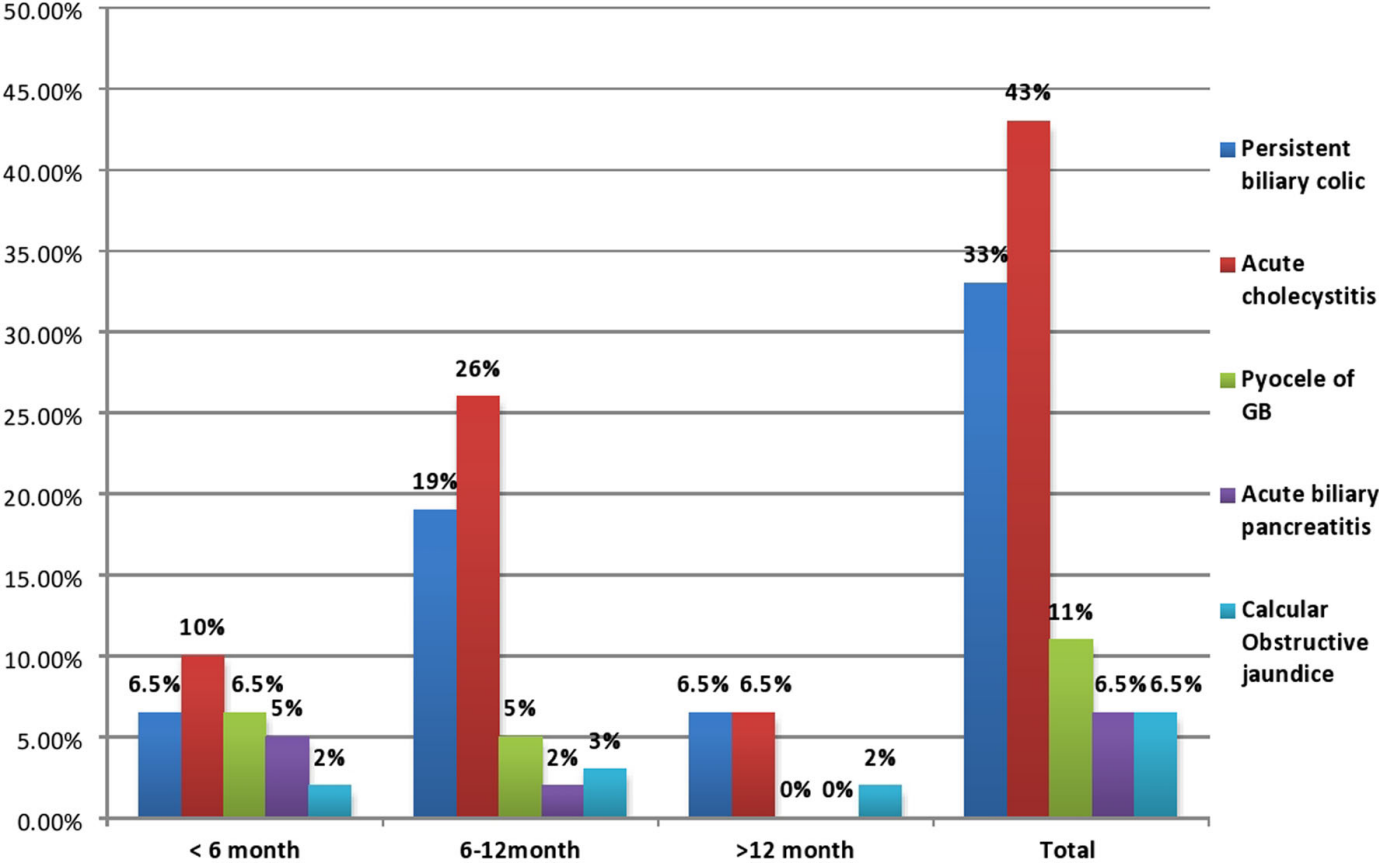
Time of surgery and diagnosis, among complicated cases of SG group (*n* = 61)

**Fig. 3:**
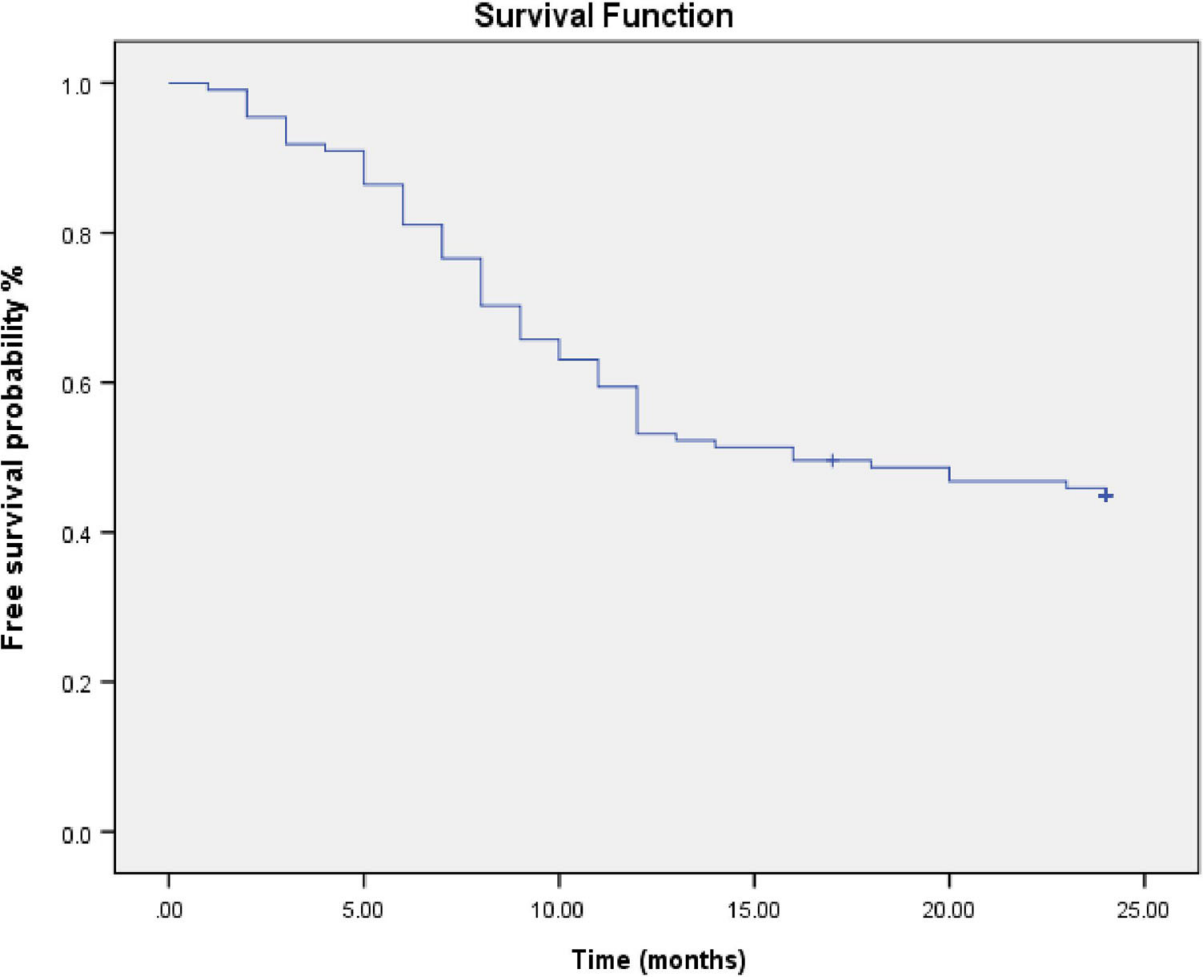
Kaplan–Meier survival estimate plot for gallstone symptom-free patients after laparoscopic sleeve gastrectomy (*n* = 111)

There was no statistically significant difference between LSG + LC group and SG group in all perioperative data except mean operative time and postoperative hospital stay, which were longer in the SG + LC group than in the SG-only group (*P* < 0.001). Most patients in both study groups were females, between 36 and 45 years, ASA II. The initial BMI and total cholesterol in SG + LC and SG groups were 42.7 ± 2.92, 43.1 ± 2.18 and 205.3 ± 13.28 mg/dl, 201.73 ± 17.07 mg/dl, respectively. Family history of GB stones was in 81 cases (36.5%) in both groups. Open conversion occurred in 3 cases (3%) in the SG + LC group and two cases (2%) in the SG group. Early postoperative complications occurred in 9 cases (9%) in the SG + LC group and in 3 cases (3%) in the SG group. Re-intervention occurred in 3 patients (3%) and one patient (1%) in SG + LC and SG groups, respectively. Late postoperative complications were in 6 patients (6%) in SG + LC and in 3 patients (3%) in the SG group where the commonest complications were incisional hernia and adhesive intestinal obstruction. Sixty-one patients (61/111, 55%) of the SG group showed gall stone symptoms requiring surgery in the follow-up period mostly presented with acute cholecystitis (26/61,42.7%), mostly diagnosed within one year after SG surgery (52/61,85%) (Table 1) (Fig. 2). The Kaplan–Meier analysis showed gallstone symptom-free rates of 91.9% at 3 months, 81.1% at 6 months, 53.2% at 12 months and 45% at 24 months after SG (Fig. 3).

**Table 1:** Demographic and perioperative outcomes among the studied groups (*n* = 222)

Demographic and peri-operative data of the studied groups (*n* = 222)				
Variables	LSG + CC group (*n* = 111)	SG group (*n* = 111)	Test	*P* value
Age (years)				
18–35	34(30.5%)	42(38%)	^a^2.295	0.317
36–45	69(62%)	65(58.5%)		
> 45	8(7.5%)	4(3.5%)		
Sex				
Male	21(19%)	25(22.5%)		
Female	90(81%)	86(77.5%)	^a^0.349	0.507
ASA				
I	11(10%)	16(14%)	^a^3.951	0.413
II	81(73%)	85(76.5%)		
III	9(8%)	5(4.5%)		
IV	5(4.5%)	3(3%)		
V	5(4.5%)	2(2%)		
Initial BMI (kg/m^2^) (mean ± SD)	42.7 ± 2.95	43.1 ± 2.18	^b^-0.620	0.536
Initial total Cholesterol (mg/dl) (mean ± SD)	205.3 ± 13.28	201.73 ± 17.07	^b^1.738	0.084
Co-morbidities				
Diabetes mellitus				
Non-insulin	19(17%)	26(23%)	^a^4.752	0.093
Insulin	11(10%)	19(17%)		
Hypertension	17(15%)	26(23%)	^a^2.336	0.126
COPD	5(4.5%)	11(10%)	^a^2.224	0.119
Family history of gall bladder stones	40(36%)	41(37%)	^a^0.019	0.889
Intraoperative outcome				
Operative time(min) (mean ± SD)	141.15 ± 2.48	100.43 ± 16.7	^a^25.44	< 0.001**
Converted lap to open	3(3%)	2(2%)	^a^0.205	0.651
Converted lap to open of splenic injury(short gastric vessel injury)	1(1%)	2(2%)		
Uncontrolled bile leakage	1(1%)	0(0%)	^a^0.0001	1.00
Extensive tissue adhesive	1(1%)	0(0%)		
None	108(97%)	109(98%)		
Postoperative outcome among the studied groups (n = 222)				
Duration of postoperative pain(days) (mean ± SD) on VAS	3.18 ± 1.08	2.93 ± 0.95	^a^1.847	0.066
Postoperative hospital stay(days) (mean ± SD)	2.21 ± 0.51	1.37 ± 0.69	^a^9.410	< 0.001**
Early postoperative complications				
Wound infection	3(3%)	2(2%)	^a^0.632	0.959
Bile leakage	1(1%)	0(0%)		
Gastric leak	1(1%)	1(1%)		
Ileus	1(1%)	0(0%)		
Pneumonia	2(2%)	0(0%)		
Bleeding	1(1%)	0(0%)		
None	102(91%)	108(97%)		
Clavien-Dindo classification				
0 I II III	102(91%) 3(3%) 3(3%) 3( 3%)	108(97%) 2(2%) 0(0%) 1(1%)	^a^1.409	0.703
Re-intervention				
Causes of re-intervention Slipped cystic artery ligature Uncontrolled bile leak Gastric leak	3(3%) 1(1%) 1` (1%) 1` (1%)	1(1%) 0(0%) 0(0%) 1(1%)	^a^1.018	0.313
Late postoperative complications				
Incisional hernia Adhesive intestinal obstruction	3(%) 3(%)	2(2%) 1(1%)	^b^1.242	0.537
Presentations of gallbladder diseases(during 2 years follow up period)				
Asymptomatic Symptomatic requiring surgery 1. Persistent biliary colic 2. Acute cholecystitis 3. Empyema of GB 4. Acute biliary pancreatitis 5. Calcular Obstructive jaundice	– – – – – –	50/111(45%) 61/111(55%) 20/61(32.8%) 26/61(42.7%) 7/61(11.5%) 4/61(6.5%) 4/61(6.5%)	–	–
Times of diagnosis of complicated gall bladder cases				
< 6 months 6–12 months > 12 months	– – –	18/61 (30%) 34/61(55%) 9/61(15%)	–	–

*LSG* + *CC* Laparoscopic sleeve gastrectomy + Concomitant cholecystectomy, *LSG* Laparoscopic sleeve gastrectomy, *SD* Standard deviation, *BMI* Body mass index, *ASA* American Society of Anesthesiologists Physical Status Scale, *COPD* Chronic obstructive pulmonary disease

^a^Chi square test (χ^2^) ^b^Independent *t*-test, * ≤ 0.05: Statistically significant, ** ≤ 0.001: Highly statistical significant

In term of changes in BMI, %EWL and cholesterol level during follow-up, there was highly statistical significant difference (≤ 0.001) between both groups as regard all values except for BMI at 3 months and year two BMI either most or least successful. BMI values at 3 months, 6 months, 1 year and 2 years were higher among SG group, and %EWL at 6 months, one year and two years was higher among SG + LC group; however, at three months it was higher among SG group. Serum cholesterol levels at 3 months, 6 months and 2 years were higher among SG + LC group. In addition, highly statistical significant difference (≤ 0.001**) was found on comparing changes of BMI, %EWL and cholesterol level inside each group (Table 2).

**Table 2:** Changes of body mass index, % of excess weight loss and cholesterol level during follow-up among the studied groups (*n* = 222)

Changes	LSG + CC group (*n* = 111) Mean ± SD	SG group (*n* = 111) Mean ± SD	^a^ Test	*P* value
Change in BMI				
Base line	42.7 ± 2.95	43.1 ± 2.18	−0.620	0.536
At 3 month	34.24 ± 1.29	34.48 ± 1.27	−1.360	0.175
At 6 months	30.49 ± 1.07	32.1 ± 1.41	−9.061	< 0.001**
1 year	24.63 ± 1.26	27.78 ± 1.23	−18.88	< 0.001**
2 years	24.77 ± 1.27	27.0 ± 1.45	−12.24	< 0.001**
	^b^ < 0.001**	^b^ < 0.001**		
Year 2 BMI				
Most successful	24.92 ± 0.77	25.09 ± 0.89	−1.394	0.165
Least successful	41.18 ± 1.83	40.37 ± 2.47	0.947	0.352
% of excess body weight loss				
3 months	20.24 ± 0.97	23.11 ± 5.39	−5.528	< 0.001**
6 months	29.50 ± 1.97	27.71 ± 4.23	4.050	< 0.001**
1 years	43.27 ± 1.93	35.70 ± 5.22	14.33	< 0.001**
2 years	43.98 ± 2.36	38.85 ± 3.82	12.04	< 0.001**
	^b^ < 0.001**	^b^ < 0.001**		
Serum cholesterol changes (mg/dl)				
Preoperative	205.3 ± 13.28	201.73 ± 17.07	1.738	0.084
3 months	200.20 ± 2.29	190.77 ± 2.67	28.25	< 0.001**
6 months	170.23 ± 4.38	163.73 ± 6.58	8.672	< 0.001**
2 years	140.47 ± 3.59	118.34 ± 7.16	29.09	< 0.001**
	^b^ < 0.001**	^b^ < 0.001**		

*LSG* + *CC* Laparoscopic sleeve gastrectomy + Concomitant cholecystectomy, *LSG* Laparoscopic sleeve gastrectomy, *SD* Standard deviation, *BMI* Body mass index

^a^Independent *t*-test, ^b^ Repeated Measures ANOVA with Paired t-test among each group, * ≤ 0.05: Statistically significant, ** ≤ 0.001: Highly statistical significant

Characteristics and Perioperative outcomes of 61 patients with complicated gallbladder stones requiring surgery after SG group (delayed LC group).

The majority of cases (47/61, 77%) were female, aged between 36 and 45 years (29/61, 49%), had ASA II (45/61, 74%), cholesterol level (208.9 ± 16.53 mg/dl) and a BMI (43.8 ± 1.84), with diabetes being the most common comorbidity (24/61, 39%) and a family history of gall stones in 54% of cases (33/61). The operative time of delayed LC after SG took 50.13 ± 1.99 min, and conversion to open cholecystectomy occurred in 2 cases (2/61, 3.2%). The average postoperative hospital stay after delayed LC was 2.28 ± 0.57 days. Early postoperative complications occurred in 4 cases (4/61, 6.4%). Re-intervention was required in two cases (2/61, 3.2%). Late postoperative complications occurred in 2 cases (2/61, 3.2%) due to incisional hernia (Table 3).

**Table 3:** Characteristics and perioperative outcomes of Group B symptomatizing patients requiring laparoscopic cholecystectomy after bariatric surgery (*n* = 61)

	(*n* = 61)
*Preoperative data*	
Age(years)	
20–35	29(48%)
36–45	30(49%)
> 45	2(3%)
Sex	
Male Female	14(23%) 47(77%)
ASA	
I II III IV V	12(20%) 45(74%) 2(3%) 1(1.5%) 1(1.5%)
BMI (kg/m2) (mean ± SD)	43.8 ± 1.84
Total Cholesterol (mg/dl) (mean ± SD)	208.9 ± 16.53
*Co-morbidities*	
Diabetes mellitus	
Non-insulin Insulin Hypertension COPD	14(23%) 10(16%) 14(23%) 6(10%)
Previous abdominal surgeries(other than LS)	
Caesarean section Appendectomy Previous hernia repair Abdominoplasty None	5(8%) 1(2%) 2(3%) 4(7%) 49(80%)
Family history of gall bladder stones	33(54%)
Intraoperative data	
Operative time in minutes (mean ± SD)	50.13 ± 1.99
Conversion	2(3.2%)
Causes of conversion	
Bile leakage Tissue adhesive at Calot triangle	1(1.6%) 1(1.6%)
Postoperative data	
Mean duration of postoperative pain (days) (mean ± SD)	2.24 ± 0.43
Average of Postoperative hospital stay(days) (mean ± SD)	2.28 ± 0.57
Early postoperative complications	
Wound infection Bile leakage Gastric leak Ileus Bleeding Pneumonia None	1(1.6%) 1(1.6%) 0(0%) 0(0%) 1(1.6%) 1(1.6%) 57(93.6%)
Clavien-Dindo classification	
0 I II III	57 (93.6%) 1(1.6%) 1(1.6%) 2(3.2%)
Re-intervention	2( 3.2%)
Causes of re-intervention	
Bile leak Bleeding due to slipped cystic artery	1(1.6%) 1(1.6%)
Late postoperative complications	
Incisional hernia Adhesive intestinal obstruction	2(3.6%) 0(0%)

*SD* Standard deviation, *BMI* Body mass index, *ASA* American Society of Anesthesiologists Physical Status Scale, *COPD* Chronic obstructive pulmonary disease

There was highly statistical significant association (≤ 0.001**) between initial BMI, initial cholesterol, family history of gallbladder stones, %EWL at 3, 6, 12 and 24 months and symptomatic gallbladder stone where patients with %EWL at one year > 37, 6 months > 28, 2 years > 38 and 3 months > 25, initial cholesterol > 200 mg/dl, + ve family history, and initial BMI > 44 kg/m^2^ were 98, 38, 36.7, 17.5, 13.9, 6.17 and 5.09 times more likely to have symptomatic gallbladder stone, respectively (Table 4).

**Table 4:** Relation between the independent factors and developing symptomatic gallstone requiring surgeries among Group B with univariate analysis (*n* = 111)

Factors	Symptomatic gall bladder stone(*n* = 61)	Asymptomatic gall bladder stone(*n* = 50)	^a^*P* value	Univariate OR(95% CI)
*Age (years)*				
20–35(*n* = 42) 36–45(*n* = 65) > 45(*n* = 4)	29(69%) 30(46%) 2(50%)	13(31%) 35(54%) 2(50%)	0.066	2.6(1.15–5.89) Ref 1.16(0.15–8.79)
*Sex*				
Male(*n* = 25) Female(*n* = 86)	14(56%) 47(55%)	11(44%) 39(45%)	0.905	1.06(0.43–2.59) Ref
Initial BMI, kg/m^2^ ≤ Median(44)(*n* = 80) > Median(44)(*n* = 31)	43.8 ± 1.84 36(45%) 25(81%)	42.04 ± 2.18 44(55%) 6(19%)	0.001**	Ref 5.09(1.88–13.76)
*%EWL*				
3 month ≤ Median(25)(*n* = 62) > Median(25)(*n* = 49) 6 month ≤ Median(28)(*n* = 60) > Median(28)(*n* = 51) 1 year ≤ Median(37)(*n* = 60) > Median(37)(*n* = 51) 2 years ≤ Median(38)(*n* = 57) > Median(38)(*n* = 54)	27.21 ± 1.88 18(29%) 43(88%) 30.93 ± 2.36 14(23%) 47(92%) 39.87 ± 2.49 12(20%) 49(96%) 41.62 ± 2.56 12(21%) 49(91%)	18.12 ± 3.81 44(71%) 6(12%) 23.78 ± 2.15 46(77%) 4(8%) 30.62 ± 2.36 48(80%) 2(4%) 35.46 ± 1.86 45(79%) 5(9%)	< 0.001** < 0.001** < 0.001** < 0.001**	Ref 17.5(6.34–48.34) Ref 38(11.8–126.1) Ref 98(20.8–461.3) Ref 36.7(12–112.5)
Initial cholesterol, mg/dl ≤ Median(200)(*n* = 56) > Median(200)(*n* = 55)	208.9 ± 16.53 15(27%) 46(84%)	192.9 ± 13.24 41(73%) 9(16%)	< 0.001 **	Ref 13.9(5.53–35.32)
Diabetes Yes(*n* = 45) No(*n* = 66)	24(53%) 37(56%)	21(47%) 29(44%)	0.777	Ref 1.12(0.52–3.39)
Family history positive*(n* = 41) negative(*n* = 70)	33(80%) 28(40%)	8(20%) 42(60%)	< 0.001**	6.17(2.49–15.35) Ref

*BMI* Body mass index, *%EWL* Excess body weight loss, *OR* Odds Ratio, *CI* Confidence Interval

^a^Chi square test (χ^2^), * ≤ 0.05: Statistically significant, ** ≤ 0.001: Highly statistical significant

The best fitting logistic regression model for determining the independent factors affecting symptomatic gallstone formation requiring surgeries among group B showed that + ve family history of gallbladder stones, initial cholesterol > 200 mg/dl, %EWL at 6 months > 28, %EWL at 1 year > 37 and %EWL at 2 years > 38 were statistically significant (*P* ≤ 0.05*) independent predictors for developing symptomatic gallstone requiring surgeries (Table 5).

**Table 5:** Multivariate regression analysis determining the independent factors affecting symptomatic gallstone formation requiring surgeries among Group B (*n* = 111)

Independent factors	B *coefficient*	S.E	*P* value	Exp(B)	95% C.I. for EXP(B)	
					Lower	Upper
+ ve family history	5.588	2.196	0.011*	267.3	3.614	19,765.7
Initial cholesterol, mg/dl > Median(200)	-2.277	1.131	0.044*	0.103	0.011	0.941
%EWL at 6 months > Median(28)	-2.963	1.354	0.029*	0.052	0.004	0.734
%EWL at 1 year > Median(37)	-4.637	1.601	0.004*	0.010	0.00	0.223
%EWL at 2 year > Median(38)	-3.582	1.496	0.017*	0.028	0.001	0.522
Constant	5.959	1.589	< 0.001**	387.11	–	–

Chi-square test for model coefficient = 124.5, *P*-value ≤ 0.001** SE: Standard Error, CI: Confidence Interval, ^*^ ≤ 0.05: Statistically significant, ** ≤ 0.001: Highly statistical significant

Variable(s) entered on equation: Initial BMI, Initial cholesterol, Family history of gall bladder stones, %EWL at 3 months, %EWL at 6 months, %EWL at 1 year, %EWL at 2 year

## Discussion

The current study concluded that simultaneous LC and LSG are preferable. Although SG with LC is associated with longer operative time and postoperative hospital stays, the incidence of SG and LC complications is approximately the same as delaying LC after SG. Concomitant cholecystectomy eliminates the need for subsequent cholecystectomy in patients with symptomatic gallbladder stones.

### Complications of SG + LC versus Delayed LC After Previous SG

Previous studies focused on operative time, intraoperative and postoperative complications, and particularly biliary complications from lengthy surgery due to simultaneous LC. Complications and length of hospital stay were not increased while concomitant LC added 40.7 min (range 15–110 min) to SG 16 patients by Coşkun et al.; when they performed LC, LSG, they added a 5 mm incision for a right hypochondrial extra trocar [8]. Others found that concomitant LC increases surgery time by 35 min in SG without increasing hospitalization days and that two patients had bile leakage: one requiring open conversion with hepatico-jejunostomy and the other stopped spontaneously [7]. Another study confirmed that concomitant LC with SG raised surgical site infection risk by 0.6% and concluded that concomitant LC and LSG are safe in gallstone disease [13].

Our series found that concomitant LC with SG increased operative time by 40.7 min which is similar to the literature but the postoperative hospital stays was increased due to increased operative time and postoperative pain duration. Open conversion due to gallbladder removal in the two cases; uncontrolled bile leakage that required conversion due to partial tear of the common bile duct repaired by simple interrupted sutures with Vicryl 4/0 sutures; and the second case, due to extensive adhesion that required open partial cholecystectomy. Furthermore, re-intervention after SG + LC occurred in 2 patients related to gallbladder surgery; one patient had slipped cystic artery bleeding, managed with re-laparoscopy, and the second patient had biliary leakage and was managed with endoscopic stenting. Furthermore, three cases (one case of LSG + CC and 2 cases of SG-only) required conversion due to bleeding from slipped short gastric vessels and required controlling by ligature. Early postoperative complications occurred in 3 cases of LSG + CC: one case had uncontrolled bile leak and controlled endoscopic stenting, the second case required laparoscopic re-intervention due to bleeding from slipped cystic artery and controlled by clipping while the last case required endoscopic gastric stenting due to gastric leak diagnosed one week after surgery by fever, tachycardia and CT with contrast. In SG group, one case complained of gastric leak and diagnosed by fever, tachycardia and CT with contrast 5 days postoperative and managed successfully by endoscopic gastric stenting. These complications were not severe, self-limited and without delayed complications. We recommend starting with SG first and then LC due to many causes: The first is SG is more time-consuming than LC and more exhausting, and the second cause is that if conversion occurred due to biliary cause, it will be through right subcostal incision, not midline incision, to deal better with biliary complications through this approach and less delayed wound complications. For Tarantino et al., starting with LC first, then SG required one additional trocar in all patients in the right upper quadrant, and they performed cholecystectomy early in operation when the surgeon's patience was still high, and the surgeon was not exhausted [14].

Delayed LC after SG may be safer due to decreasing the fat content of the abdominal cavity, which helps to identify distinct anatomy. However, Papavramidis et al. in their study on delayed LC after bariatric surgery did not agree with this concept and showed that conversion to an open procedure in six patients (17.6%) was due to severe adhesions in four patients (11.7%) and massive bleeding from the gallbladder bed in two patients (5.9%). The mean operative time of this series was 75 ± 12 min, and the mean hospitalization was 2.8 ± 1.1 days. They recommended LC + SG at the same time [15]. Conversion during delayed LC occurred in 2 cases (2/61,3.2%) due to bile leak that was not detected by laparoscope and, after conversion; injury to the common hepatic duct was diagnosed and treated by hepatico-jejunostomy, while conversion in the other case was due to extensive adhesion in the Calot triangle that required conversion and treatment by subtotal cholecystectomy. In our study, intraoperative bleeding was not a cause of conversion. early postoperative bile re-intervention occurred in two cases in delayed LC; one case due to bile leak and required endoscopic stenting while the other cases required laparoscopic re-intervention due to bleeding slipped cystic artery and required clip controlling. In our study, intraoperative bleeding was not a cause of conversion.

**Fig. 4:**
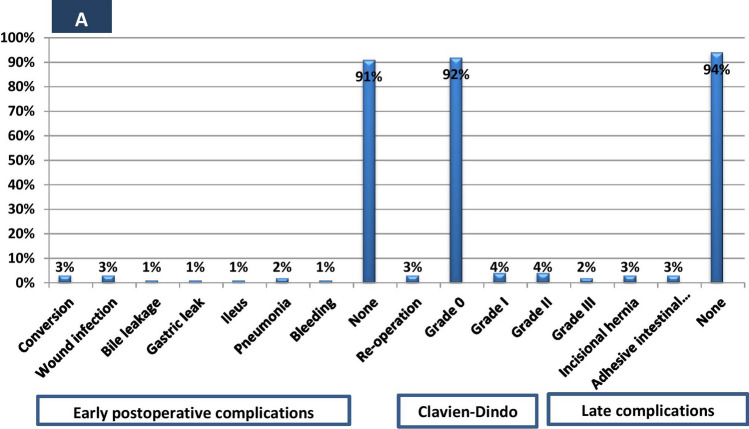
Frequency of intraoperative and postoperative outcomes among SG + LC group (*n* = 111)

**Fig. 5:**
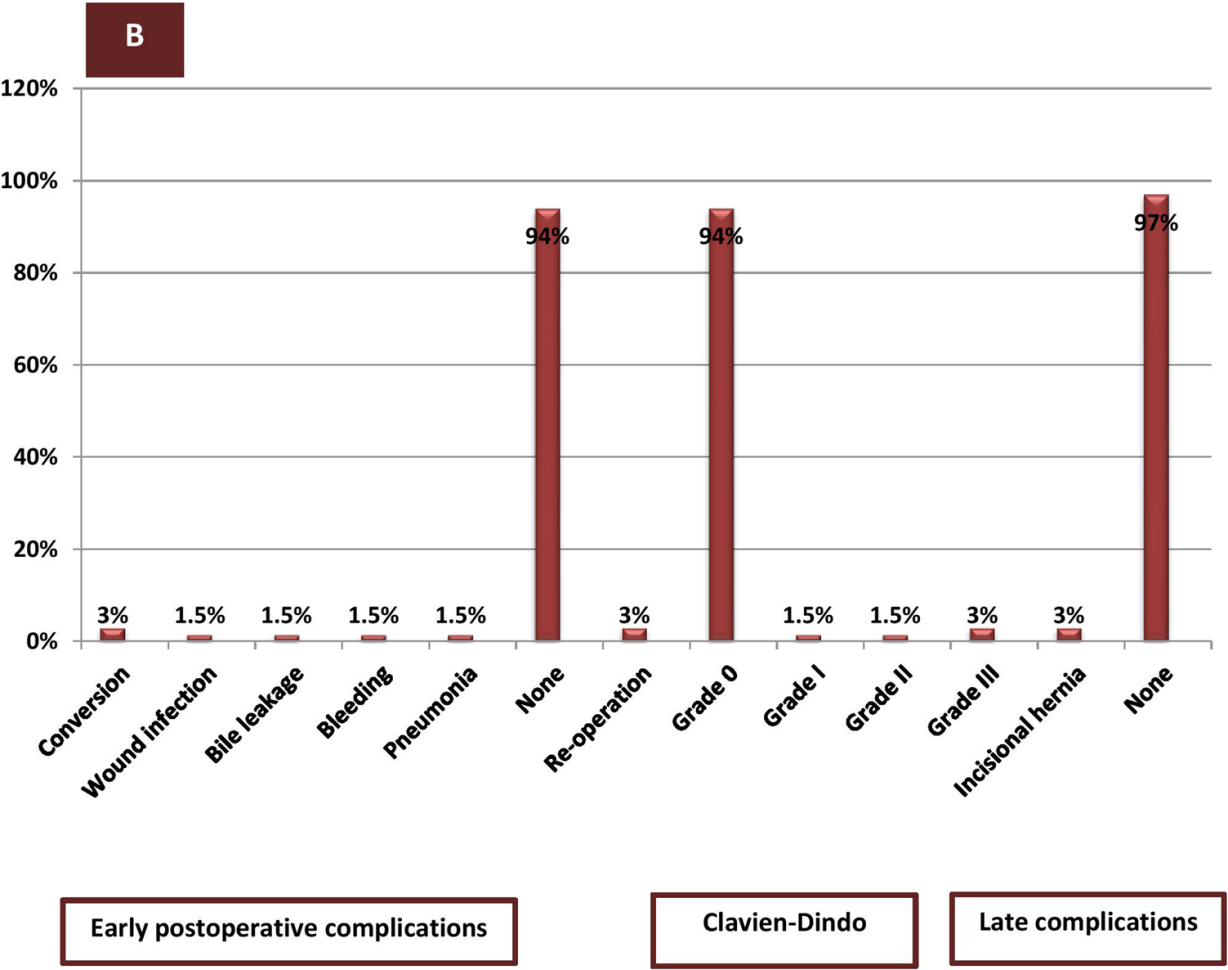
Frequency of intraoperative and postoperative outcomes among delayed LC group (*n* = 61)

When comparing perioperative outcomes and surgical risks between LSG + LC and delayed LC, our result stated that operative time for concomitant LC (40.7 min) is shorter than when performing delayed LC (50.13 ± 1.99) after SG, mostly due to the time taken for trocar insertion and not related to fat loss. Weight loss did not make delayed LC easier, but complications may occur. The postoperative hospital stays in delayed LC is nearly the same as in LSG + LC (2.28 ± 0.57 vs. 2.21 ± 0.51), while the duration of postoperative pain was longer in LSG + LC than in delayed LC (3.18 ± 1.08 vs. 2.24 ± 0.43). We found that incidence of conversion due to biliary complications were nearly identical in both groups and the causes of conversion due to biliary leak was severer in delayed LC than concomitant LC. The incidence of early postoperative complications is higher in LSG + LC than in LC alone (9/111, 9% vs. 4/61, 6.4%) however, most of them were due to wound infection, pneumonia and ileus with improvement by conservative treatment. The variation in the number of patients in both groups is an additional factor (Figs. 4, 5).

### Incidence, Timing and Forms of Complicated GB Stones After SG Without LC

Different studies stated a high incidence of symptomatic gallbladder stones after SG and recommended performing LC together with SG. Sioka et al. validated that 13.0% of patients who had evident cholelithiasis preoperatively developed complicated cholelithiasis. They concluded that routine concomitant LC could be considered because the proportion of patients who developed complications, especially those with potentially significant morbidities, is high, the actual technical difficulties during subsequent cholecystectomy and the time to develop complications is short [16]. In addition, Papavramidis et al. reported that 34 patients (40.5 percent of cases) had symptomatic cholelithiasis within 2 years of bariatric surgery. Eight (23.5%) developed the condition within 6 months, 13 (36.1%) within developed it 12 months, and the remaining 13 (36.1%) developed it within 24 months post-bariatric surgery. Acute cholecystitis affected 20 patients (58.2%). Also, two patients (5.9%) developed choledocholithiasis with obstructive jaundice, requiring endoscopic retrograde cholangiopancreatography, sphincterotomy and gallstone extraction prior to surgery. According to their results, SG + LC is the preferred technique SG + LC is the preferred technique [15].

Others found a decreased incidence of symptomatic Gallstones and did not propose routine LC. Raziel et al. [7] found that of 43 patients with asymptomatic gallstones, four (9.3%) developed symptoms, two of which were acute cholecystitis, one pancreatitis and one empyema of the gallbladder. Another study by Tsirline et al. [17] found that 7.8% of asymptomatic individuals had cholecystectomy after bariatric surgery.

Our study found a higher frequency of symptomatic Gallstones after SG than prior studies with 61 patients (61/111, 55%) requiring another LC procedure and most cases operated within a year of SG (52/61, 85%). The higher incidence is due to the higher sample size, significant weight loss within 1 year and consistent follow-up with close observation and ultrasonography that detects many cases of symptomatic gall stones. Our commonest indication for LC after SG was acute cholecystitis not improving with conservative treatment and required surgery within 72 h followed by persistent biliary colic. Other authors concur with our assessment that urgent LC is the optimum treatment for biliary colic because delayed LC increases readmission, operative time, hospital stay and conversion rate compared to urgent LC cases [18].

We found four cases of calcular obstructive jaundice (3.5%), three of which responded to conservative treatment with ciprofloxacin antibiotics and analgesics, and one of which required ERCP to remove a big stone (10 mm). During ERCP, no difficulty was encountered in identifying major duodenal papillae or canulation.

In this study, % EWL and family history were risk factors for developing symptomatic gallstones requiring surgery in SG group. Rapid weight loss causes bile to become saturated with cholesterol, causing gallbladder stones to symptomatize. According to Hemminki et al. 36% of patients with gallstone disease have a family history of gallstone stones [19].

Limitation:

A small sample size to evaluate safety of SG and LC, but comprehensive data assembling with high resolution overcomes it. Bigger sample size is also required to assess both groups' safety accurately.

## Conclusion

The incidence of gall stones following SG is significant, exposing some patients to another procedure, stress and anesthesia risk. Concomitant LC during SG is safe and practical in obese patients with asymptomatic gallstones.

## Data Availability

All relevant raw data will be freely available to any researcher wishing to use them for non-commercial purposes without breaching participant confidentiality. The datasets generated during and/or analyzed during the current study are available from the corresponding author on reasonable request.
